# Delayed Chemotherapy-Induced Nausea – A Nurse-Led International Observational Study in Routine Oncology Practice (CINrate)

**DOI:** 10.1177/23779608251398116

**Published:** 2025-12-02

**Authors:** Ramona Engst, Agnes Glaus, Ulrike Moessner, Stefan Ott, Antje Koller

**Affiliations:** 1 School of Health, 112888 OST Eastern Switzerland University of Applied Sciences, St.Gallen, Switzerland; 2 Tumor and Breast Center ZeTuP St.Gallen, Switzerland; 3 221229 University Medical Center, Freiburg, Germany

**Keywords:** Delayed chemotherapy-induced nausea, prevalence, supportive care, oncology nursing, survey

## Abstract

**Background:**

Nausea and vomiting negatively impact patients’ quality of life and may influence systemic antitumour therapy (CHT). While delayed CHT-associated nausea (dCIN) is most commonly linked to moderate or highly emetogenic regimens, evidence suggests that it may also occur with low (LEC) and minimally emetogenic CHT (MinEC). This study aims to assess the occurrence and characteristics of dCIN in real-world clinical settings, with an emphasis on patients receiving LEC and MinEC.

**Methods:**

In a prospective multicentre international cross-sectional study, adult oncology outpatients receiving systemic antitumour therapy rated the intensity of dCIN daily on a 0–100 visual analogue scale (VAS) for five consecutive days. The primary endpoint was dCIN occurrence in LEC and MinEC. Secondary endpoints included known risk factors and the relationship between dCIN and patient characteristics.

**Results:**

Among 172 patients, 65 (38%) received LEC and 31 (18%) MinEC. Most patients received antiemetic therapy in accordance with MASCC/ESMO and ASCO guidelines. dCIN occurred in 18.5% (n = 12; 95% CI [10.5, 29.1]) of LEC and 3% (n = 1; 95% CI [0.04, 14.1]) of MinEC patients. Only 3 patients (1.7%) reported vomiting. Younger age and gastrointestinal tumours were independent risk factors for dCIN. Emetogenicity of therapy, fear, and prior CHT-associated vomiting did not remain significant in the model.

**Conclusions:**

A considerable proportion of patients receiving LEC still experience dCIN in real-world clinical settings. These findings highlight the need for improved symptom management and tailored interventions beyond traditional emetogenic risk classification.

The trial was registered at clinicaltrials.gov NCT04342780 (Date of registration: 03/25/2020).

## Introduction

Nausea and vomiting rank among the most feared side effects of chemotherapy (CHT)^1^ (Spichiger et al., 2011). Since the introduction of efficacious antiemetics such as 5-HT3 receptor antagonists (5-HT3RA) in the late 1990s and Neurokinin receptor antagonists (NK1-RA) in the early 2000s, the occurrence of nausea and vomiting has decreased markedly in clinical practice (Jordan et al., 2015). Advances in understanding the pathophysiology of CHT-associated^2^ nausea and vomiting (CINV) along with the identification of patient-specific risk factors, have contributed substantially to this progress (Dranitsaris et al., 2017). In recent years, strategies for antiemetic prophylaxis have increasingly recognized that nausea and vomiting arise from a complex, multifactorial process involving numerous neurotransmitters and receptors.

## Review of the Literature

The widely used abbreviation ‘CINV’ reflects the historical view of nausea and vomiting as closely related symptoms in the context of CHT. Today, CHT-associated nausea (CIN) is recognized as a phenomenon related to, but distinct from CHT-associated vomiting (CIV), with its own pathophysiology and potential therapeutic approaches (Grunberg, 2012).With the appropriate use of evidence-based antiemetic regimens, CIV can be prevented in most patients, while CIN is less well controlled (Jordan et al., 2015). Firstly, the variance may derive from the differences in pathophysiology and a limited efficacy of common antiemetics on CIN. Secondly, vomiting is an observable event, while nausea is a sensation that is more difficult to quantify and may appear in conjunction with or independent of vomiting (Grunberg, 2012). The subjective nature of nausea, that makes it difficult to observe from the outside, contributes to the inconsistency in the assessment of nausea between patients and health care professionals (Wild et al., 2005). This highlights the important role of nurses in facilitating accurate symptom evaluation by fostering open communication and applying structured assessment methods. Nausea occurring more than 24 h after systemic antitumour therapy administration is defined as delayed CIN (dCIN). As patients are less well monitored after the administration of CHT is finished, dCIN may be more problematic than acute CIN (aCIN) (Escobar et al., 2015). Nursing support in patient education, symptom monitoring, and early intervention remains essential during this vulnerable phase.

CIN treatment is based on well-known guidelines, for example by the Multinational Association for Supportive Care in Cancer, the S3 guideline ‘Supportive Therapy of the German Guideline Program in Oncology’ or the guidelines of the American Society of Clinical Oncology (ASCO) (Deutsche Krebsgesellschaft, Deutsche Krebshilfe, AWMF [Leitlinienprogramm Onkologie], 2017; Hesketh et al., 2017; MASCC/ESMO, 2016; National Comprehensive Cancer Network [NCCN], 2019). CIN management consists of individualized pharmacological regimens aimed at both prevention and rescue treatment of breakthrough CIN. Breakthrough CIN is defined as the occurrence of CIN after the use of guideline-compliant prophylactic antiemetics (Escobar et al., 2015; Molassiotis et al., 2008). The focus is on CIN prevention, as it is more difficult to treat CIN once it has occurred (Grunberg, 2012). In these guidelines, treatment is based on the emetogenicity of systemic antitumour therapy agents. This is divided into four groups (high, moderate, low, and minimally emetogenic). Notably, the data base for high and moderate emetogenic chemotherapies is very good to good. However, the recommendations for low and minimally emetogenic chemotherapies are based on moderate to poor data (Deutsche Krebsgesellschaft, Deutsche Krebshilfe, AWMF [Leitlinienprogramm Onkologie], 2017). For the prophylaxis of CIN in low-emetogenic treatments, the CINV guidelines recommend the use of a single agent on the day of CHT (e.g., dexamethasone OR Metoclopramide OR 5-HT3 receptor antagonist), in minimal no routine prophylaxis is recommended (Deutsche Krebsgesellschaft, Deutsche Krebshilfe, AWMF [Leitlinienprogramm Onkologie], 2017; Hesketh et al., 2017; MASCC/ESMO, 2016; NCCN, 2019).

As studies on dCIN occurrence mostly come from randomized clinical pharmaceutical trials, data on the clinical ‘real-life’ occurrence of dCIN are scarce (Molassiotis et al., 2008). Risk factors for dCIN include emetogenicity of the chemotherapeutic drug. Although dCIN affects up to 60% of patients receiving moderately to highly emetogenic CHT, data for low-emetogenic CHT are limited, data are poorer for those with low-emetogenic CHT. However, there is evidence that dCIN still affects about 20% of patients in low-emetogenic therapy (Molassiotis et al., 2008).

To improve the understanding of the occurrence of CIN in clinical practice, the identification of risk factors is vital (Dranitsaris et al., 2017; Molassiotis et al., 2014). Apart from the emetogenicity of the antitumour medication, risk factors include clinical factors such as type of tumour diagnosis or previous CHT, demographic factors such as lower age, or female gender, as well as psychological factors such as fear of CHT. Patients who previously experienced nausea (e.g., during pregnancy, while travelling or in the context of previous CHT applications) are at higher risk of developing CIN. The cumulative number of prior CHT cycles appears to increase CIN risk for CIN, while regular overconsumption of alcohol appears to be a protective factor (Molassiotis et al., 2014). However, data on risk factors specifically for dCIN are scarce.

**Objectives:** This nurse-led survey aimed to assess the occurrence of dCIN in routine clinical practice with emphasis on low-emetogenic CHT and to describe the antiemetic treatment and characteristics of patients with dCIN.

## Methods

### Design, Setting and Sample

In this binational prospective multicentre cross-sectional study, patients reported dCIN as well as associated clinical and demographic factors. Patients were recruited in the oncology outpatient clinics of three accredited cancer centres in Switzerland and Germany. Participants were enrolled on the first day of a new cycle of any systemic antitumour therapy. Patients were assessed for sufficient proficiency in German. At enrolment, each participant signed an informed consent form.

Exclusion criteria were cognitive impairment, which could significantly interfere with consent or participation as assessed by health care professional or research assistants, concomitant radiotherapy, pre-existing nausea in the previous two days, or previous participation in CINrate (each person participated only once). Participants hospitalized within five days of systemic antitumour therapy administration were excluded. Data collection was planned for three to ten weeks depending on the context in each setting.

### Variables and Measurement

The primary endpoint was *dCIN*. Secondary endpoints were *aCIN*, *CIV*, *antiemetic prescription* (prophylactic and rescue antiemetic medication). In accordance with Glaus et al., the occurrence of dCIN was evaluated using self-report diaries with visual analogue scales (VAS). It was measured daily on a scale from 0 mm (no nausea) to 100 mm (most severe nausea imaginable) for five days after the last day of systemic antitumour therapy. The VAS is a suitable tool for assessing nausea intensity in patients and has been shown to be sensitive to changes over time (Börjeson et al., 1997). The five days following the last day of systemic therapy were defined as days 1 to 5 to account for variations in the duration of different treatment regimens (Glaus et al., 2004). Patients were instructed to place a vertical mark on the line corresponding to their level of nausea. In addition, participants recorded time and duration of each nausea episode. The *occurrence of dCIN* was counted if the VAS score exceeded 5 mm. The occurrence of *significant dCIN* was counted if the VAS score exceeded 25 mm (Glaus et al., 2004). Patients were instructed to complete the VAS directly after each episode, but at least once daily in the evening before going to bed. aCIN was defined as nausea reported within the first 24 h after administration of CHT, whereas dCIN was defined as nausea reported from 24 h onward (Glaus et al., 2004). Vomiting was assessed using an analogous VAS (0–100 mm; 0 = no vomiting; 100 = worst imaginable vomiting). Patients noted each episode of vomiting. They also recorded the date, time, and type of each antiemetic medication (prescribed or alternative) they took for nausea or vomiting within the days recorded. Distress from dCIN was measured daily on a 4-point scale (0 = no distress, 3 = high distress).

Demographic and clinical data included variables known to be risk factors for developing CIN. These included *age*, *sex*, *marital status*, *fear of CHT*, and *sleep quality and duration* the night before recruitment. Clinical data included *type of diagnosis*, *comorbidities* (e.g., diabetes, cardiovascular disease, gastrointestinal (GI) disease, musculoskeletal syndrome, thyroid disorders), metastases, months since diagnosis, *previous cancer therapies*, *history of CINV* in previous cycles, history of *motion sickness* or *nausea gravidarum*, and *alcohol consumption* (Bush et al., 1998). The emetogenicity of systemic antitumour therapy protocols was determined using the MASCC/ESMO, 2016 classification in combination with the S3 guideline ‘Supportive Therapy of the Oncology Guideline Program’ (Deutsche Krebsgesellschaft, Deutsche Krebshilfe, AWMF [Leitlinienprogramm Onkologie], 2017). If the antitumour drug was not listed in the MASCC/ESMO classification and the S3 guideline, the ASCO and NCCN 2019 guidelines were considered (Hesketh et al., 2017; NCCN, 2019). In case of combined antitumour drugs, the drug with the highest degree of emetogenicity was considered. In the rare cases where the guideline classifications differed, the higher level of emetogenicity was chosen (e.g., Alemtuzumab in ASCO and MASCC ‘moderate’, and in NCCN ‘minimal’).

### Data Analysis

Data were pooled across the three study sites. Occurrence of the primary and secondary endpoints, as well as demographic and clinical variables, were analysed descriptively. Occurrence and 95% confidence intervals were calculated for primary and secondary outcomes. Bivariate group differences between dCIN and no dCIN were tested for each risk factor with the Mann-Whitney-U-Test or a Fisher's exact test as appropriate based on data scales under consideration. The relationship between dCIN and risk factors was determined using a stepwise multiple logistic regression model. A type I error threshold of .05 was applied for all statistical tests. For logistic regression, variables were selected through stepwise inclusion based on p-value thresholds and theoretical relevance. Risk factors were included in the model if the bivariate comparison or multicollinearity analysis suggested that they could contribute to the explanatory power of the model (Table 1). Contrasts were analysed to assess the impact of categorical variables. Missing values were handled by multiple imputation. While the variables age, diagnosis, and emetogenicity were almost completely available, the variables CIN during previous CHT and fear of CHT had 15% and 26% missing values, respectively. Therefore, missing value imputation was performed using chained equations (MICE) with the variables CIN during previous CHT and fear of CHT as dependent variables and the other variables mentioned above, as well as gender, as independent variables. The necessary and plausible assumption of missingness at random (MAR) is fulfilled. Due to the many missing values, 40 imputations were performed, and the results were merged in SPSS according to Rubin's rule. In addition, a descriptive post hoc power analysis was performed (α = .05, two-sided) for the evaluation of the primary outcome (dCIN) using logistic regression. Data analysis was performed using IBM SPSS statistics vs. 28. The study was conducted in accordance with the Declaration of Helsinki and was approved by the Ethics Committee of Eastern Switzerland (BASEC Nr. 2020-01115 EKOS 20/081) and by the Ethics Committee Freiburg i.Br. (Nr. 21-1019). Written informed consent was obtained from all participants prior to inclusion in the study.

**Table 1.** Clinical and Demographic Characteristics and Their Distribution in Relation to Delayed Nausea.

Variables	Number of Participants (n)	All (N = 172)	Delayed Nausea (n = 31)	No Delayed Nausea (n = 141)	p
Center; % (n)	Center 1	26.2 (45)	11.1 (5)	88.9 (40)	0.169^+^
	Center 2	54.7 (94)	23.4 (22)	76.6 (72)	
	Center 3	19.1 (33)	12.1 (4)	87.9 (29)	
Age	Mean (median); min/max	62.9 (64.2); 25/86	57.3 (58.1); 25/81	64.1 (65.2); 35/86	0.027°
Gender; % (n)	Female	42.4 (73)	20.5 (15)	79.5 (58)	0.548^+^
	Male	57.6 (99)	16.2 (16)	83.8 (83)	
Diagnosis Groups; % (n)	GI^a^ tumors	30.8 (53)	34.0 (18)	66.0 (35)	0.002^+^
	Non-GI solid tumors	51.2 (88)	12.5 (11)	87.5 (77)	
	Hematological tumors	18.0 (31)	6.5 (2)	93.5 (29)	
Comorbidity; % (n)	Yes	82.0 (141)	17.7 (25)	82.3 (116)	0.800^+^
Metastasis; % (n)	Yes	58 (94)	18.1 (17)	81.9 (77)	0.758^+^
Months since diagnosis	Mean (median); min/max	39.7 (17); 1/289	26.9 (10.0); 1/161	42.6 (18); 1/289	0.059°
Emetogenicity level; % (n)	Minimal	18.0 (31)	3.2 (1)	96.8 (30)	0.023^+^
	Low	37.8 (65)	18.5 (12)	81.5 (53)	
	Moderate	35.5 (61)	23.0 (14)	77.0 (47)	
	High	8.7 (15)	26.7 (4)	73.3 (11)	
Previous antitumour therapy; % (n)^b^	Chemotherapy	57.3 (98)	16.3 (16)	83.7 (82)	0.549^+^
	Radiotherapy	35.5 (61)	21.3 (13)	78.7 (48)	0.414^+^
	Immunotherapy	14.5 (25)	8.0 (2)	92.0 (23)	0.258^+^
	Hormone therapy	15.1 (26)	11.5 (3)	88.5 (23)	0.421^+^
Live alone in household; % (n)	Yes	22.1 (38)	26.3 (10)	73.7 (28)	0.153^+^
Performance status (ECOG)^c^	Mean (median); 25/75 percentile	1.2 (1); 1/2	1.2 (1.0); 1/2	1.9 (1.0); 1/2	0.975°
Fear of systemic antitumour therapy^e^	Mean (median); 25/75 percentile	19.1 (6.2); 0/33.3	29.4 (22.4); 2.3/58.9	16.5 (4.2); 0/24	0.027°
Hours of sleep before day of systemic antitumour therapy	Mean (median); 25/75 percentile	6.7 (7); 6/7.5	6.4 (6.0); 5.5/7	6.6 (7); 6/7.6	0.122°
Sleep quality before day of systemic antitumour therapy^d^	Mean (median); 25/75 percentile	43.9 (44.4); 16.4/71	40.4 (45.0); 20/64	44.6 (43.8); 14.8/74.5	0.573°
Travel sickness; % (n)	Yes	19.8 (34)	29.4 (10)	70.6 (24)	0.079^+^
Past systemic antitumour therapy; % (n)		84.9 (146)	17.8 (26)	82.2 (120)	
Nausea during past systemic antitumour therapy; % of above (n)	Yes	48.6 (71)	26.8 (19)	73.2 (52)	0.009^+^
Women with Kids; %, n		35.3 (59)	18.6 (11)	81.4 (48)	
Nausea during pregnancy; % of above (n)	Yes	62.7 (37)	18.9 (7)	81.0 (30)	0.956^+^
Alcohol consumption^f^	Mean (median); 25/75 percentile	2 (2); 1/3	2.2 (2); 1/3	1.9 (2); 1/3	0.371°

a GI Gastrointestinal; ^b^Percentages do not sum up to 100 because of overlap between the categories; ^c^ECOG performance status: 0 (fully active) to 4 (completely disabled; totally confined to bed or chair); ^d^Scale 0 (excellent) to 100 (miserably); ^e^Scale 0 (no fear) to 100 (greatest possible fear); In one center, fear was not surveyed (all n = 101; delayed nausea n = 26); ^f^Scale 0 (never) to 4 (more than 4 times a week) ^+^Fisher's exact test; °Mann-Whitney-U-Test.

## Results

**Figure 1.**
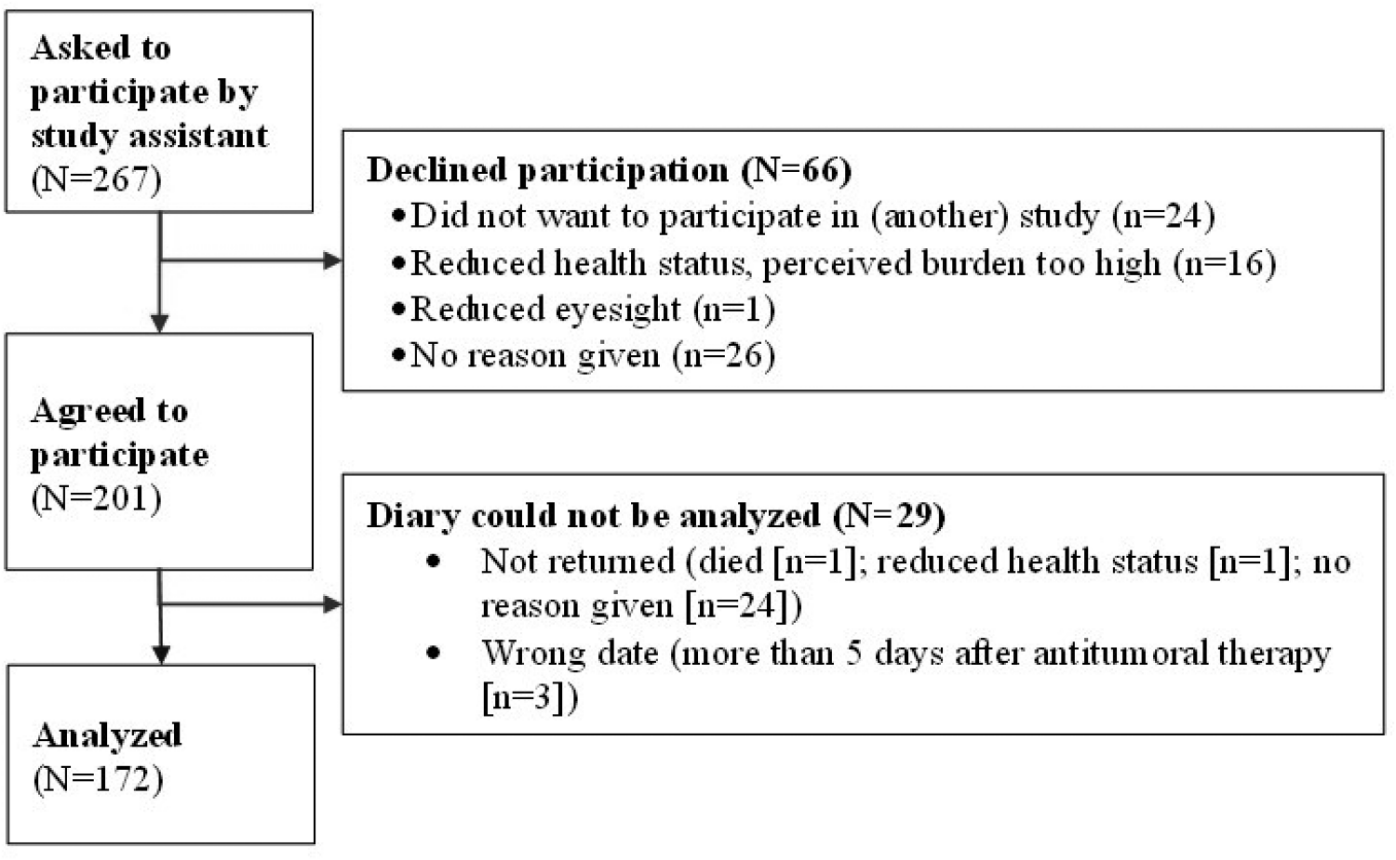
Flow Chart of Recruitment and Data Collection.

Across the three centres, 201 patients were recruited from June 2020 to May 2022 (recruitment rate 75%). Two centres were located in the German-speaking part of Switzerland and one in southern Germany. A total of 267 patients were approached, of whom 201 agreed to participate, resulting in a response rate of approximately 75%. Of the diaries distributed, 26 were not returned, and in 3 cases the completion date did not match the date of CHT administration. Consequently, a total of 172 diaries were included in the final analysis (Figure 1).

### Sample

Of the 172 participants who completed the diary, 42% (n = 73) were women. The mean age was 63 years (range 25 to 86; Table 1). The distribution of diagnoses varied across centres depending on their respective treatment focus. Diagnoses were classified into three groups: GI malignancies (31%, n = 53) included colon, pancreas, oesophagus and gallbladder cancer. Non-GI solid tumours (51%, n = 88) included breast, lung, prostate, ovarian and urothelial carcinomas. Haemato-oncological diagnoses (18%, n = 31) included myelomas, leukaemias and lymphomas. In total, the 172 patients received over 50 different antitumour drugs in varying dosages, combinations, and routes of administration. Low-emetogenic antitumour regimens consisted of a mean of 1.5 (median = 1; range = 1–5), minimally emetogenic antitumour regimens of 1.3 drugs per patient (median = 1, range = 1-3). Low-emetogenic drugs included (38%, n = 65) taxanes (e.g., paclitaxel) antimetabolites (e.g., gemcitabine), topoisomerase-inhibitors (i.e., etoposide), immunotherapeutics (e.g., atezolizumab), proteasome-inhibitors (e.g., bortezomib), angiogenese-inhibitors (i.e., aflibercept), and antracyclines (e.g., liposomale doxorubicin). Minimally emetogenic drugs (18%, n = 31) included monoclonal antibodies (e.g., trastuzumab, rituximab), checkpoint inhibitors (e.g., pembrolizumab), vinca alkaloids (i.e., vinorelbine), and other agents (e.g., bleomycin) (Table 2). Antiemetic regimens are presented in Table 3.

**Table 2.** Antitumour Drugs That Were Scored With the Highest Emetogenicity in the Protocol (N=172).

		Center 1 (n = 45)	Center 2 (n = 94)	Center 3 (n = 33)	All (n = 172)
Minimal; n	Pembrolizumab	5	4		9
	Nivolumab	3	3		6
	Rituximab	2	2		4
	Trastuzumab	3			3
	Atezolizumab		2		2
	Bevacizumab	1	1		2
	Vinorelbin	1			1
	Daratumumab	1			1
	Ramucirumab		1		1
	Obinutuzumab		1		1
	Bleomycine			1	1
Sum minimal		16	14	1	31
Low; n	Paclitaxel	9	4	6	18
	Gemcitabine	1	8	4	13
	Docetaxel	5	1	2	8
	Etoposid	1	4		5
	Pemetrexed			3	3
	Carfilzomib		3		3
	Cabazitaxel	1		1	2
	Bortezomib	1	1		2
	liposomale Doxorubicin		1	1	2
	Trastzumab-emantasine	2			2
	Atezolizumab			1	1
	Elotuzumab		1		1
	Aflibercept		1		1
	Decitabine		1		1
	Inotuzumab Ozogamicin		1		1
	Brentuximab		1		1
Sum low		**20**	**27**	**18**	**65**
Moderate; n	Oxaliplatin	1	15	2	18
	Carboplatin	2	8	7	17
	Irinotecan	2	11	2	15
	Bendamustin		4	1	5
	Cyclophosphamid	1	4		5
	Arsenic trioxide			1	1
Sum moderate		6	42	13	61
High; n	A&C (Adriamycin & Cyclophosphamid)	2	6	1	9
	Cisplatin	1	5		6
Sum high		3	11	1	15

**Table 3.** Antiemetic Regimen in Patients Receiving low or Minimal Emetogenic Chemotherapy.

Guideline Conform n (%)	Antiemetic Therapy and Prophylaxis	Emetogenicity of Systemic Therapy According to Guidelines	
		Minimal (n = 31)	Low (n = 65)
Less than guidelines recommend	No antiemetic medication	0	7 (11%)
As guidelines recommend	No antiemetic medication	26 (84%)	0
	Dex	2 (6%)	35 (54%)
	Dex + 5-HT3	0	1 (1.5%)
	5-HT3	0	1 (1.5%)
More than guidelines recommend	Dex	3 (10%)	0
	Dex + 5-HT3	0	15 (23%)
	Dex + 5-HT3 + Dex d1 + 2(+3 + 4)	0	1 (1.5%)
	Dex + 5-HT3 + Prednisone continuous therapy	0	3 (4.5%)
	Dex + 5-HT3 + Dex d1 + 2 + 21	0	2 (3%)

Dex: Dexamethasone; 5-HT3: 5-HT3 receptor antagonists; d: day.

### Occurrence, Intensity, and Distress of dCIN

The occurrence of dCIN in low-emetogenic CHT was 18.5% (n = 12 of n = 65; 95% CI [10.5, 29.1]), in minimally emetogenic CHT 3% (n = 1 of n = 31; 95% CI [0.04, 14.1]). Overall, 18% of the participants (n = 31, 95% CI [12.8, 24.3]) experienced dCIN (VAS > 5), while 12% of the patients (n = 20) experienced significant dCIN (VAS > 25;Table 2).

The mean intensity of dCIN was 33.3 (range = 10.8–83.3 on the VAS 0-100). Mean intensity of dCIN in patients who experienced significant dCIN was 42.6 (range = 13.7–83.3). Mean distress by dCIN was 1.4 (range = 0 [none] - 3.0 [high]) in patients with dCIN > 5 and 1.5 (range = 0.5–3.0) in patients with significant dCIN (Table 4).

**Table 4.** dCIN Occurrence, Intensity, and Distress Compared Across Emetogenicity Level of Antitumour Drug (N=172).

Emetogenicity of Antitumour Drug	dCIN Occurrence % (95% CI; n)	dCIN Intensity; Mean (min/max)	dCIN >25 Occurrence; % (n)	dCIN Distress Mean (min/max)
Minimal (n = 31)	3.2 (0.4-14.1; 1)	76.0 (76/76)	3.2 (1)	2 (2/2)
Low (n = 65)	18.5 (10.5–29.1; 12)	31.5 (12.5/60.8)	12.3 (8)	1.7 (0.5/3.0)
Moderate (n = 61)	23.0 (13.8–36.6; 14)	32.8 (11.1/83.3)	13.1 (8)	1.5 (1.0/2.2)
High (n = 15)	26.7 (9.7–51.7; 4)	29.4 (10.8/41.2)	20 (3)	1.0 (1.0/1.0)

Legend: dCIN = delayed chemotherapy associated nausea; CI = confidence-interval.

### CIN Trajectories

In sum, n = 34 (19.8%, 95% CI [14.3, 26.2]) patients experienced CIN within five days of CHT. Thirteen patients (8%) reported aCIN (VAS > 5). Of these, 3 (23%) experienced only aCIN, whereas 10 also experienced dCIN on the following days. Five patients (38.5%) experienced significant aCIN (VAS > 25). On average, aCIN started 4 h after CHT (range = 0–9 h) and lasted about 6 h (range = 0.33–14). The mean intensity of aCIN was 28.7 (range = 5–78.4 [VAS 0-100]). The mean intensity of significant aCIN was 48.8 (range = 30–78.4).

On average, dCIN started after 27 h (range = 1–78 h) and lasted on average 17 h (range = 0.5–68.5 h). In those patients with CIN, dCIN started on day 1 following CHT in 11 participants (32%), and in 7 participants (21%) on day 2. In 3 participants (9%) dCIN occurred on day 3 following CHT.

None of the participants developed dCIN after day 3. The risk of developing nausea was highest on day 0 (13 of 172 = 7.6%) and decreased each day (day 1: 11 of 172 = 6.4%; day 2: 7 of 172 = 4.1%, day 3: 3 of 172 = 1.7%).

CIN occurred on average over 2.7 days, persisting until day 5 in 4 individuals (29%). Because the diary concluded on day 5, the duration of dCIN beyond this period could not be determined for these participants.

### Vomiting: Overall Occurrence, Intensity, and Distress

Vomiting occurred in 3 male patients that were treated in one centre. Two patients had colon cancer and one oesophageal cancer. The mean intensity was 23.6 (range = 10.8–50.0 on the VAS 0-100;) and the mean distress was 1.4 (range = 1-2 on the scale 0 [none] to 3 [high]). The range of episodes per day was 1 to 4.

### Regression Analysis

For regression analysis a 2-step approach was used. In a first step, of all measured risk factors, 5 factors identified by bivariate analyses were included in the logistic model (age, diagnosis [summarized in three groups: solid, GI, haematological], emetogenicity of CHT, fear of CHT, CIN during previous CHT [Table 1]). In the stepwise logistic regression analysis, 3 risk factors showed potential explanatory power, i.e., diagnoses, age and CIN during previous CHT, whereas emetogenicity of CHT and fear of CHT were excluded from the model due to the lack of explanatory power. In the second step, the remaining risk factors were applied to 40 instances of the data set, generated by missing value imputation, using a fixed logistic regression model. The results were consolidated using Rubin's rule.

In summary, CIN during previous CHT proved to be insignificant (p = .110) due to the large variation between instances, younger age proved to be trending towards significance (i.e., occurrence of dCIN tends to decrease with increasing age, p = .055), while the diagnosis showed significant differences. dCIN was significantly higher for GI tumours than for solid (p = .004) and haematological malignancy (p = .010; Table 5). It should also be noted that significant variables show high power (e.g., β = .821 for diagnosis GI vs. solid), while no significant correlation could be found when power was too low (e.g., β = .160 for diagnosis hema vs. solid).

**Table 5.** Pooled Logistic Regression on dCIN (N = 172).

Predictor	B (SE)	OR	95% CI (OR)	*p*	Power β
Prior nausea with CHT	0.818 (0.512)	2.267	0.830–6.192	.110	.359
Age (years)	−0.033 (0.017)	0.968	0.936–1.001	.055	.485
Diagnosis: GI vs. solid	1.311 (0.455)	3.709	1.521–9.047	.004	.822
Diagnosis: hema. vs. solid	−0.778 (0.812)	0.459	0.093–2.257	.338	.160
Diagnosis: hema vs. GI	−2.089 (0.806)	0.124	0.026–0.601	.010	.736

*Notes.* OR = odds ratio; CI = confidence interval. Estimates pooled using Rubin's Rules across the multiple imputations.

## Discussion and Analysis

This real-world multicentre study shows that dCIN occurs in nearly 20% of patients receiving LEC, despite the use of antiemetic regimens mostly consistent with international guidelines (Hesketh et al., 2017; Deutsche Krebsgesellschaft, Deutsche Krebshilfe, AWMF [Leitlinienprogramm Onkologie]; MASCC/ESMO, 2016; NCCN, 2019). This confirms that LEC cannot be considered clinically negligible in terms of nausea risk.

Traditional predictors such as CHT emetogenicity did not remain in the final model, suggesting a need to move beyond classification-based risk assessments. Younger age and GI tumour diagnoses were stronger predictors of dCIN, consistent with findings by Hayashi et al. (2018), who also identified prior CINV and performance status as relevant factors. While the sample in the present study showed some overlap, performance status did not remain significant, possibly due to the high functioning level in the outpatient population. Compared to Hayashi et al. (2018) Klicken oder tippen Sie hier, um Text einzugeben., the study included a wider mix of therapies including monoclonal antibodies and modern immunotherapeutics. While the heterogeneity of treatments may limit comparability with more controlled trials, it enhances the external validity of the findings by capturing current clinical practice. Therefore, the findings of the present study expand on prior research by emphasizing the persistence of dCIN in a real-world, guideline-compliant setting, even among therapies traditionally considered ‘low-risk”.

Since most patients experienced dCIN within 3 days after CHT, closer monitoring and follow-up of patients receiving LEC and MinEC is recommended. As patients are discharged shortly after treatment and thus beyond clinical observation during the delayed phase, digital tools or scheduled outreach (e.g., day-2 calls) may support earlier symptom detection and improve outcomes.

Fortunately, vomiting does not seem to play a major role compared with past times. In early 2000, Glaus et al. reported an occurrence rate of vomiting of 13% (n = 31) for acute emesis and 38% (n = 93) in the delayed phase (Glaus et al., 2004). In this survey, vomiting occurred in only three participants (1.7%) who all had GI tumours. Inflammatory processes in the GI tract are suspected of having an unfavourable effect on nausea (Chen et al., 2022). Therapeutic concepts should therefore address potential additional risk factors and different pathophysiologies of dCIN compared with aCIN or with emesis. Options may include integrative therapeutic approaches, such as dietary measures or exercise interventions (Gala et al., 2022; Herrstedt et al., 2024). Further research is warranted to explore additional factors contributing to dCIN and to refine risk-stratification strategies for better tailored antiemetic interventions (Chen et al., 2022). Younger individuals and those diagnosed with GI cancers appeared to be at higher risk for experiencing dCIN (Li et al., 2022).

### Strengths and Limitations

Limitations of the present study include its cross-sectional design and lack of long-term follow-up. The absence of a formal a priori power calculation, due to the exploratory nature of the study, may limit the precision of subgroup analyses. The study was conducted only in three accredited centres in the German-speaking regions of Switzerland and Southern Germany, which may limit the generalizability of the findings. Patients differed slightly across centres, for instance, one centre included more patients with haematological malignancies, and minor variations in centre-specific practices, such as antiemetic use, were observed. These differences reflect the heterogeneity inherent in routine clinical practice. Additionally, data collection occurred partially during the COVID-19 pandemic, and the impact of wearing masks could not be excluded. However, the risk of bias introduced by mask-wearing seems minimal as there was no significant difference in CIN rates observed between the three centres with different mask wearing routines. Although the study population was relatively small and sample sizes differed between centres, the calculated confidence intervals account for this fact. Some centres had slightly modified treatment standards, by tending to be more generous in the use of additional reserve antiemetics. However, the rationale for deviations from the guidelines could not always be interpreted appropriately and therefore was not included in the analysis.

A significant strength of the study lies in its representation of real-world clinical practice. In the meantime, new versions of the guidelines have been published, as these are regularly updated (MASCC/ESMO, 2023; NCCN, 2025). The authors are confident that the findings remain broadly applicable and can be interpreted in the context of current recommendations.

### Implications for Practice

The quantity and variability of antitumour regimens applied routinely in oncology day clinics are substantial. Nurses, in particular, face challenges in managing the complexity and sheer volume of these therapies, ensuring accurate monitoring, tailored patient education, and timely identification of adverse effects. Now that common risk factors such as the emetogenicity of antitumour therapy no longer provide sufficient evidence to prevent delayed nausea in the remaining 20% of patients, it is a major challenge for nurses to carry out targeted symptom management with appropriate patient education. The present study underscores the need to explore new feasible treatment strategies to effectively address the remaining fifth of affected individuals in the demanding routine of everyday clinical practice (Alderman et al., 2022; Li et al., 2022). Health care professionals should address dCIN in all patients receiving CHT while they are still within reach during day-clinic visits. Nurses play a pivotal role in the early identification and management of dCIN by systematically assessing patients’ prior experiences with nausea, monitoring risk factors such as age, diagnosis, and previous CIN, and utilizing structured patient diaries or checklists in routine assessments. Practical strategies include proactive patient education on anticipatory symptoms, guidance on the timing and adherence to antiemetic medication, and supportive counselling to manage anxiety and expectations. Tailoring interventions to individual patient needs and maintaining open communication channels for symptom reporting can help nurses to target care more effectively and enhance patients’ self-management at home. However, further development and implementation of integrative treatment approaches are needed, that are both effective and safe for home use, to ensure optimal self-management of dCIN (Gala et al., 2022).

### Conclusions

This study demonstrates that delayed nausea remains clinically relevant even in patients receiving low- and minimally emetogenic CHT. Given the limitations of current emetogenic classifications in predicting dCIN, a broader risk assessment model incorporating patient-specific and treatment-contextual factors may be indicated. Nurses should be aware that ‘low-risk’ does not equal ‘no-risk’ and implement proactive symptom monitoring and patient education strategies to support early detection and management of dCIN.

## Notes

1. Whenever possible, the term “chemotherapy (CHT)” is used for consistency and clarity throughout the manuscript. We acknowledge that “antitumour therapy” may be considered a more contemporary term; however, CHT is retained here due to its widespread use in common abbreviations, such as chemotherapy-induced nausea (CIN).

2. As numerous risk factors beyond the emetogenicity of chemotherapy have been identified, the term “chemotherapy-associated nausea” would be more accurate than “chemotherapy-induced.” However, the abbreviation CIN is retained to ensure consistency and comparability with previous research.

## References

[bibr1-23779608251398116] Alderman B. Hui D. Mukhopadhyay S. Bouleuc C. Case A. A. Amano K. Crawford G. B. Feo G. de Sbrana A. Tanco K. To J. Garsed J. Davis M. (2022). Multinational association of supportive care in cancer (MASCC) expert opinion/consensus guidance on the use of cannabinoids for gastrointestinal symptoms in patients with cancer. Supportive Care in Cancer : Official Journal of the Multinational Association of Supportive Care in Cancer, 31(1), 39. 10.1007/s00520-022-07480-x 36525085

[bibr2-23779608251398116] Börjeson S. Hursti T. J. Peterson C. Fredikson M. Fürst C. J. Avall-Lundqvist E. Steineck G. (1997). Similarities and differences in assessing nausea on a verbal category scale and a visual analogue scale. CANCER NURSING, 20(4), 260–266. 10.1097/00002820-199708000-00005 9265812

[bibr3-23779608251398116] Bush K. Kivlahan D. R. McDonell M. B. Fihn S. D. Bradley K. A. (1998). The AUDIT alcohol consumption questions (AUDIT-C): An effective brief screening test for problem drinking. Ambulatory care quality improvement project (ACQUIP). alcohol use disorders identification test. Archives of Internal Medicine, 158(16), 1789–1795. 10.1001/archinte.158.16.1789 9738608

[bibr4-23779608251398116] Chen W. Zhao Y. Dai Y. Nie K. (2022). Gastrointestinal inflammation plays a critical role in chemotherapy-induced nausea and vomiting. European Journal of Pharmacology, 936, 175379. 10.1016/j.ejphar.2022.175379 36356927

[bibr5-23779608251398116] Deutsche Krebsgesellschaft, Deutsche Krebshilfe, AWMF (Hrsg.). (2017). *Supportive Therapie bei onkologischen PatientInnen - Langversion 1.1* [Registernummer: 032/054OL]. https://www.leitlinienprogramm-onkologie.de/leitlinien/supportive-therapie

[bibr6-23779608251398116] Dranitsaris G. Molassiotis A. Clemons M. Roeland E. Schwartzberg L. Dielenseger P. Jordan K. Young A. Aapro M. (2017). The development of a prediction tool to identify cancer patients at high risk for chemotherapy-induced nausea and vomiting. Annals of Oncology : Official Journal of the European Society for Medical Oncology, 28(6), 1260–1267. 10.1093/annonc/mdx100 28398530 PMC5452068

[bibr7-23779608251398116] Escobar Y. Cajaraville G. Virizuela J. A. Álvarez R. Muñoz A. Olariaga O. Tamés M. J. Muros B. Lecumberri M. J. Feliu J. Martínez P. Adansa J. C. Martínez M. J. López R. Blasco A. Gascón P. Calvo V. Luna P. Montalar J. Tornamira M. V. (2015). Incidence of chemotherapy-induced nausea and vomiting with moderately emetogenic chemotherapy: ADVICE (actual data of vomiting incidence by chemotherapy evaluation) study. Supportive Care in Cancer, 23(9), 2833–2840. 10.1007/s00520-015-2809-3 26081597 PMC4519584

[bibr8-23779608251398116] Gala D. Wright H. H. Zigori B. Marshall S. Crichton M. (2022). Dietary strategies for chemotherapy-induced nausea and vomiting: A systematic review. Clinical Nutrition (Edinburgh, Scotland), 41(10), 2147–2155. 10.1016/j.clnu.2022.08.003 36067586

[bibr9-23779608251398116] Glaus A. Knipping C. Morant R. Böhme C. Lebert B. Beldermann F. Glawogger B. Ortega P. F. Hüsler A. Deuson R. (2004). Chemotherapy-induced nausea and vomiting in routine practice: A European perspective. Supportive Care in Cancer, 12(10), 708–715. 10.1007/s00520-004-0662-x 15278682

[bibr10-23779608251398116] Grunberg S. (2012). Patient-centered management of chemotherapy-induced nausea and vomiting. Cancer Control, 19(2 Suppl), 10–15. 10.1177/107327481201902s03 22488023

[bibr11-23779608251398116] Hayashi T. Shimokawa M. Matsuo K. Miyoshi T. Toriyama Y. Yokota C. Taniguchi J. Hanada K. Tsumagari K. Okubo N. Koutake Y. Sakata K. Kawamata Y. Goto T. Tsurusaki Y. Koyabu M. (2018). Risk factors for delayed chemotherapy-induced nausea and vomiting with low-emetic-risk chemotherapy: A prospective, observational, multicenter study. Cancer Management and Research, 10, 4249–4255. 10.2147/CMAR.S176574 30323680 PMC6177523

[bibr12-23779608251398116] Herrstedt J. Clark-Snow R. Ruhlmann C. H. Molassiotis A. Olver I. Rapoport B. L. Aapro M. Dennis K. Hesketh P. J. Navari R. M. Schwartzberg L. Affronti M. L. Garcia-Del-Barrio M. A. Chan A. Celio L. Chow R. Fleury M. Gralla R. J. Giusti R. Scotté F. (2024). 2023 MASCC and ESMO guideline update for the prevention of chemotherapy- and radiotherapy-induced nausea and vomiting. ESMO open, 9(2), 102195. 10.1016/j.esmoop.2023.102195 38458657 PMC10937211

[bibr13-23779608251398116] Hesketh P. J. Kris M. G. Basch E. Bohlke K. Barbour S. Y. Clark-Snow R. A. Danso M. A. Dennis K. Dupuis L. L. Dusetzina S. B. Eng C. Feyer P. C. Jordan K. Noonan K. Sparacio D. Somerfield M. R. Lyman G. H. (2017). Antiemetics: American society of clinical oncology clinical practice guideline update. Journal of Clinical Oncology, 35(28), 3240–3261. 10.1200/JCO.2017.74.4789 28759346

[bibr14-23779608251398116] Jordan K. Jahn F. Aapro M. (2015). Recent developments in the prevention of chemotherapy-induced nausea and vomiting (CINV): A comprehensive review. Annals of Oncology : Official Journal of the European Society for Medical Oncology, 26(6), 1081–1090. 10.1093/annonc/mdv138 25755107

[bibr15-23779608251398116] Li K. Cai Y. Xie S. Zhou Y. Dong J. Zhu Q. Zhang J. Qiu X. (2022). Evidence summary for nonpharmacological management of chemotherapy-induced nausea and vomiting. BioMed Research International, 2022, 4741193. 10.1155/2022/4741193 36467880 PMC9712000

[bibr16-23779608251398116] MASCC/ESMO. (2016). *Antiemetic guidelines*. https://mascc.org/resources/mascc-guidelines/?refer=antiemetics-study-group

[bibr17-23779608251398116] MASCC/ESMO. (2023). *Antiemetic guidelines*. https://mascc.org/resources/mascc-guidelines/?refer=antiemetics-study-group

[bibr18-23779608251398116] Molassiotis A. Aapro M. Dicato M. Gascon P. Novoa S. A. Isambert N. Burke T. A. Gu A. Roila F. (2014). Evaluation of risk factors predicting chemotherapy-related nausea and vomiting: Results from a European prospective observational study. Journal of Pain and Symptom Management, 47(5), 839–848.e4. 10.1016/j.jpainsymman.2013.06.012 24075401

[bibr19-23779608251398116] Molassiotis A Stricker C. T. Eaby B. Velders L. Coventry P. A. (2008). Understanding the concept of chemotherapy-related nausea: The patient experience. European Journal of Cancer Care, 17(5), 444–453. 10.1111/j.1365-2354.2007.00872.x 18637116

[bibr20-23779608251398116] National Comprehensive Cancer Network (Hrsg.). (2019). *NCCN clinical practice guidelines in oncology: Antiemesis*. https://www.nccn.org/professionals/physician_gls/PDF/antiemesis.pdf

[bibr21-23779608251398116] National Comprehensive Cancer Network (Hrsg.). (2025). *NCCN Clinical Practice Guidelines in Oncology: Antiemesis*. https://www.nccn.org/professionals/physician_gls/pdf/antiemesis.pdf

[bibr22-23779608251398116] Spichiger E. Muller-Frohlich C. Denhaerynck K. Stoll H. Hantikainen V. Dodd M. (2011). Prevalence of symptoms, with a focus on fatigue, and changes of symptoms over three months in outpatients receiving cancer chemotherapy. *Swiss Med Wkly.* Vorab-Onlinepublikation. 10.4414/smw.2011.13303 22065282

[bibr23-23779608251398116] Wild D. Grove A. Martin M. Eremenco S. McElroy S. Verjee-Lorenz A. Erikson P. (2005). Principles of good practice for the translation and cultural adaptation process for patient-reported outcomes (PRO) measures: Report of the ISPOR task force for translation and cultural adaptation. Value in Health : The Journal of the International Society for Pharmacoeconomics and Outcomes Research, 8(2), 94–104. 10.1111/j.1524-4733.2005.04054.x 15804318

